# Evaluation of the Effectiveness of the Socket Preservation Technique Using Allogeneic and Xenogeneic Materials: A Randomized Controlled Trial

**DOI:** 10.3390/jfb17060262

**Published:** 2026-05-29

**Authors:** Piotr Wróbel, Magdalena Jędzierowska, Adam Piecuch, Michał Bąk, Jakub Adamczyk, Piotr Pławecki, Piotr Mojżesz, Kacper Wachol, Sylwia Wójcik, Martin Starosta, Armand Cholewka, Tadeusz Morawiec

**Affiliations:** 1 Department of Oral Surgery, Faculty of Medical Sciences in Zabrze, Medical University of Silesia, 15 Poniatowskiego Street, 40-055 Katowice, Poland; jakub.adamczyk@sum.edu.pl (J.A.); piotr.plawecki@sum.edu.pl (P.P.); piotr.mojzesz@sum.edu.pl (P.M.); kacper.wachol@sum.edu.pl (K.W.); swojcik@sum.edu.pl (S.W.); tmorawiec@sum.edu.pl (T.M.); 2 Private Dental Practice Comfortmed, 12 Wspólna Street, 44-240 Żory, Poland; mbak@sum.edu.pl; 3 Institute of Biomedical Engineering, Faculty of Science and Technology, University of Silesia in Katowice, ul. Będzińska 39, 41-200 Sosnowiec, Poland; 4 Department of Histology and Cell Pathology, Faculty of Medical Sciences in Zabrze, Medical University of Silesia in Katowice, 19 Jordana Str., 41-808 Zabrze, Poland; apiecuch@sum.edu.pl; 5 Department of Children’s Maxillofacial Surgery, Faculty of Medical Sciences in Katowice, Medical University of Silesia, 15 Poniatowskiego Street, 40-055 Katowice, Poland; 6 Department of Dentistry, Faculty of Medicine, University of Ostrava, Syllabova 19, 703 00 Ostrava-Jih, Czech Republic; martin.starosta@osu.cz; 7 Faculty of Science and Technology, University of Silesia, 75 Pułku Piechoty 1A, 41-500 Chorzów, Poland; armand.cholewka@us.edu.pl

**Keywords:** allograft, biomaterial, bone augmentation, deproteinized bovine bone, extraction, socket preservation

## Abstract

Background: The socket preservation technique involves filling the bone defect that occurs after a tooth is extracted with bone substitute material. This procedure helps to minimize bone resorption of the alveolar ridge following extraction. Various bone substitute biomaterials can be used for augmentation, including autogenous, allogeneic, and xenogeneic options. This study aimed to assess changes in alveolar ridge dimensions and variations in radiographic bone density in sockets grafted with two distinct biomaterials. Furthermore, bone biopsies collected from the grafted sites were subjected to histological analysis. Methods: Forty generally healthy patients were enrolled in the study and split into four equal groups. The first and third groups underwent first or second maxillary premolar extraction and received treatment with an allogeneic material (BIOBank®, Biobank, Paris, France), while the second and fourth groups underwent first or second mandibular molar extraction and were treated with a xenogeneic material (Geistlich Bio-Oss®, Geistlich Pharma AG, Wolhusen, Switzerland). Following tooth extraction, the appropriate biomaterial was inserted into the socket. It was covered with a collagen membrane (Geistlich Bio-Gide®, Geistlich Pharma AG, Wolhusen, Switzerland) and stabilized with sutures, which were removed seven to ten days after the procedure. Micro-CBCT scans were conducted to evaluate the dimensions of the alveolar ridge and the radiographic density of the grafted socket at 7–10 days and 6 months after the procedure. A bone trepanobiopsy was performed concurrently with implant placement six months after socket preservation. The obtained biopsy was analyzed histologically using hematoxylin and eosin (H&E) and Masson’s trichrome staining. Results: There was no statistically significant difference in alveolar ridge height or width preservation between allogeneic and xenogeneic biomaterials. After six months of healing, sockets grafted with both materials exhibited greater radiographic bone density, with significantly greater density observed in the xenograft group. Conclusions: The findings of this study suggest that the two biomaterials are comparable in their effectiveness at maintaining the dimensions of the alveolar ridge. However, the quality of the newly formed bone may differ depending on the type of biomaterial used.

## 1. Introduction

The present study is a continuation of the authors’ pilot study, which indicated differences in the healing of alveolar sockets treated with socket preservation using xenogeneic and allogeneic materials [1]. In the previous study, each of the two groups consisted of five patients and included only upper first and second premolars. Due to the small sample size, it is necessary to conduct studies on a larger group to obtain reliable results.

Although the healing process of the post-extraction socket and the characteristics of biomaterials used in socket preservation procedures were described in detail in the previous article, it is essential to briefly describe the rationale for using biomaterials in alveolar ridge preservation technique [1].

Tooth extraction results in a bone defect, which is gradually filled by newly formed bone tissue during the subsequent healing phases [2]. During the healing process, the alveolar ridge is reduced in both vertical and horizontal dimensions [3]. The use of bone substitute biomaterials aims to limit alveolar ridge resorption through osteogenic, osteoinductive, and osteoconductive effects [4,5,6]. This creates more favorable conditions for subsequent implant treatment.

The only biomaterial that possesses all of the characteristics described above is autogenous bone. Its use requires the creation of an additional surgical access site for harvesting. This characteristic, combined with its faster resorption rate compared to other materials and its limited availability, means that it is not commonly used for socket preservation technique [6,7,8].

Xenogeneic and alloplastic materials exhibit only osteoconductive properties [9]. Allografts may additionally exhibit osteoinductive properties; however, this depends on how they are processed, as demineralization exposes the collagen matrix containing bone morphogenetic proteins while simultaneously reducing the graft’s volumetric stability and potentially accelerating the remodeling of the graft into new bone tissue [9,10,11]. All three types of biomaterials described are successfully used in alveolar ridge preservation procedures [6,12,13].

Once the bone defect has been filled with a graft, various techniques are employed to seal the socket. The classic method involves achieving primary wound closure, either with or without the use of barrier membranes, to prevent the ingrowth of soft tissue and promote bone growth [4,14,15,16]. However, this may adversely affect the width of the keratinized gingiva [17].

The open healing protocol with secondary wound healing minimizes the need for soft tissue grafting and reduces the invasiveness of the procedure by limiting the need for mucoperiosteal flap preparation [16,18]. In this procedure, non-resorbable membranes, resorbable membranes, platelet rich fibrin (PRF), free gingival grafts, and medical cyanoacrylate adhesive can be used to secure the alveolar socket [16,19,20,21,22,23,24,25,26,27,28].

Radiographic bone density can be evaluated using radiographic imaging techniques and is one of the key factors influencing the initial stability of the implant and the risk of early failure [29,30,31,32]. Preoperative assessment of local bone density allows for proper planning of the implant procedure, influencing the type of implant used, the need for bone osseodensification or under-preparation of the osteotomy, and helps determine the healing time required to achieve osseointegration [29,33]. The use of alveolar preservation technique may result in increased radiographic bone density in the augmented area compared to natural healing [34,35].

The use of bone substitute biomaterials in alveolar ridge preservation procedures results in the formation of tissue containing vital bone, connective tissue, and particles of the residual graft [36,37]. Some studies indicate that as the amount of residual graft increases, the amount of vital bone decreases, which reduces bone-to-implant contact [38,39,40,41]. Although many studies indicate the therapeutic success of implants placed in augmented areas, further research is needed on the impact of residual graft material on implant treatment [42,43,44,45].

Clinical studies indicate potential differences in the maintenance of alveolar ridge dimensions, residual graft content in the alveolar socket, and bone density in the regenerated area when different materials are used in the socket preservation technique [13,34,41,46,47,48,49]. For this reason, further research is needed on the efficacy of biomaterials used in augmentation procedures.

The socket preservation technique is particularly indicated for alveolar sockets with a buccal bone plate thickness of less than 1 mm and in cases of bone fenestration or dehiscence, as such sockets are prone to greater bone resorption, resulting in significant loss of alveolar ridge width and height [50,51,52,53,54,55]. A beneficial but less significant effect of alveolar ridge preservation (ARP) was also observed in cases involving a thicker buccal bone plate [50,55]. For this reason, to obtain reliable results, we included in the present study upper first and second premolars and lower first and second molars with a buccal bone plate measuring no more than 1 mm in width.

The primary objective of the study was to determine the efficacy of allogeneic and xenogeneic materials used in socket preservation procedures. A secondary objective was to determine the radiographic density of the augmented areas using CBCT scans and to conduct a histological analysis of the collected samples.

## 2. Materials and Methods

### 2.1. Patient Selection and Allocation

**Figure 1:**
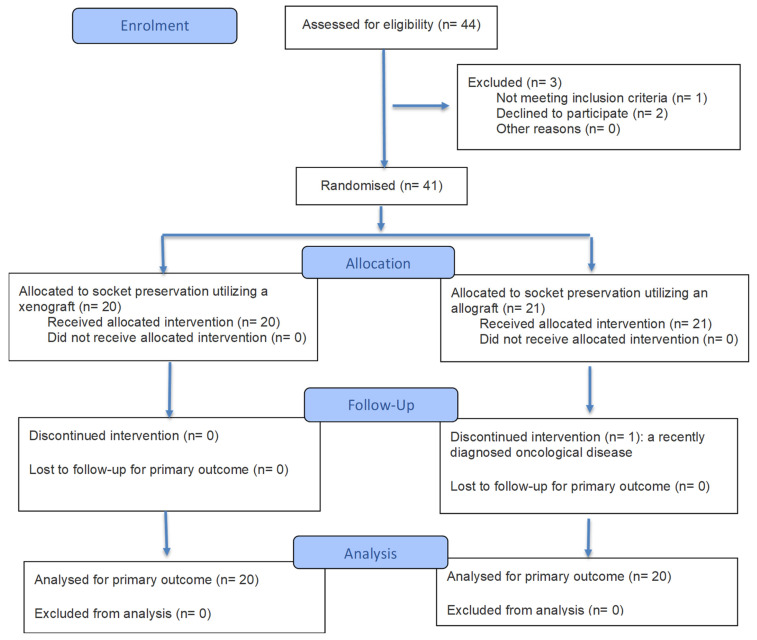
CONSORT flow diagram.

This prospective, randomized, two-arm, parallel-group clinical study was conducted on 20 patients requiring extraction of the first or second maxillary premolar and 20 patients requiring extraction of the first or second mandibular molar. Patients of both sexes were included in this study. Participant enrolment, intervention allocation, follow-up, and data analysis are represented in the CONSORT flow diagram (Figure 1). The patients were divided into 4 groups based on the type of tooth being extracted and the augmentation material used. Groups 1 and 2 consisted of alveolar sockets following first or second maxillary premolar extractions, while groups 3 and 4 consisted of alveolar sockets following first or second mandibular molar extractions. In groups 1 and 3, the augmentation material was a partially demineralized bone matrix allograft (BIOBank®, cortico-cancellous bone powder; 0.5 mm granules, Biobank, Paris, France), and in groups 2 and 4, a deproteinized bovine bone matrix (DBBM) of cortico-cancellous xenograft was used (Geistlich Bio-Oss®; 0.25–1 mm granules, Geistlich Pharma AG, Wolhusen, Switzerland). In all patients, a collagen membrane (Geistlich Bio-Gide®, Geistlich Pharma AG, Wolhusen, Switzerland) was used to cover the augmentation material. Patient randomization was performed using Research Randomizer software (https://www.randomizer.org) (accessed on 1 August 2024).

**Figure 2:**
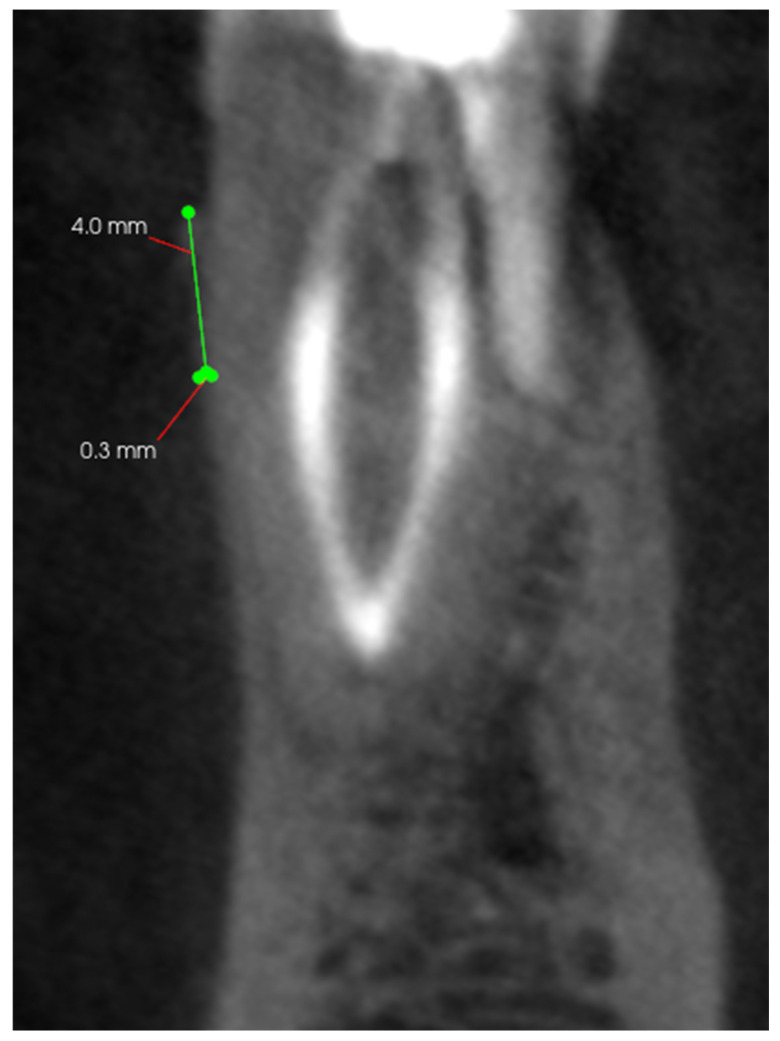
The measurement of buccal bone plate thickness.

The width of the buccal bone plate was assessed based on a preoperative micro-CBCT scan (Carestream CS 8100 3D, Carestream Dental, Atlanta, GA, USA). For maxillary premolars, the measurement was taken at the midpoint of the vestibular root width. For mandibular molars, the measurement was taken at the midpoint of the mesial root width. In both groups, the width of the vestibular lamina was measured 4 mm from the alveolar crest (Figure 2).

The inclusion criteria were a first or second maxillary premolar or first or second mandibular molar unsuitable for restorative treatment; a vestibular lamina width of less than 1 mm; age between 18 and 65 years; and the absence of vestibular lamina defects, such as fenestrations or dehiscences.

The exclusion criteria were very high or high risk drinking level according to WHO (more than 40g of ethanol per day for women and 60g of ethanol per day for men) [56], tobacco use within the last 12 months, immunosuppressive medications, systemic diseases that could affect the healing of soft or hard tissues, chemotherapy or radiation therapy to the head and neck region within the past 5 years, periodontal disease resulting in bone loss, poor oral hygiene, and extensive tooth loss.

All study participants received appropriate information about the project and provided informed consent. Approval from the local ethics board was obtained for this study (approval number: BNW/NWN/0052/KB1/43/24; date: 21 May 2024). Additionally, the clinical study was registered in the ClinicalTrials.gov registry (NCT07565558). The research was conducted in accordance with the CONSORT Reporting Guidelines [57].

The clinical part of this study was conducted at the “Comfortmed” dental clinic in Żory and the Department of Oral Surgery of the Medical University of Silesia in Bytom.

### 2.2. Sample Size Calculation and Study Design

An a priori sample size for a two-tailed independent *t*-test was calculated utilizing G*Power software (version 3.1, Heinrich-Heine-Universität Düsseldorf, Düsseldorf, Germany). The power analysis parameters were based on data from other studies on alveolar ridge preservation [58,59,60,61,62,63]. With an alpha level of 0.05, 80% statistical power and an effect size of 1.0, the required sample size was determined to be 17 patients per group. To account for potential losses during the study period, recruitment was increased to 20 participants per group.

All surgical procedures, including extraction, socket preservation, and implant placement, were performed by the same experienced oral surgeon (P.W.) to minimize inter-operator variability. Due to the nature of the procedure, the operator was not blinded to the biomaterial used during the surgery. Radiographic measurement of alveolar ridge dimensions was conducted by an independent, experienced oral surgeon examiner (M.B.), who was blinded to the grafting material used and was not involved in the surgical procedures. The bone radiographic density measurements were performed by an experienced engineer specializing in biomedical engineering and the analysis and processing of biomedical images (M.J.), blinded to the graft used in ARP. The processing of the collected bone biopsies and their histological evaluation were performed by an experienced biologist (A.P.), who was blinded to the biomaterial used.

### 2.3. Extraction with Alveolar Ridge Preservation

**Figure 3:**
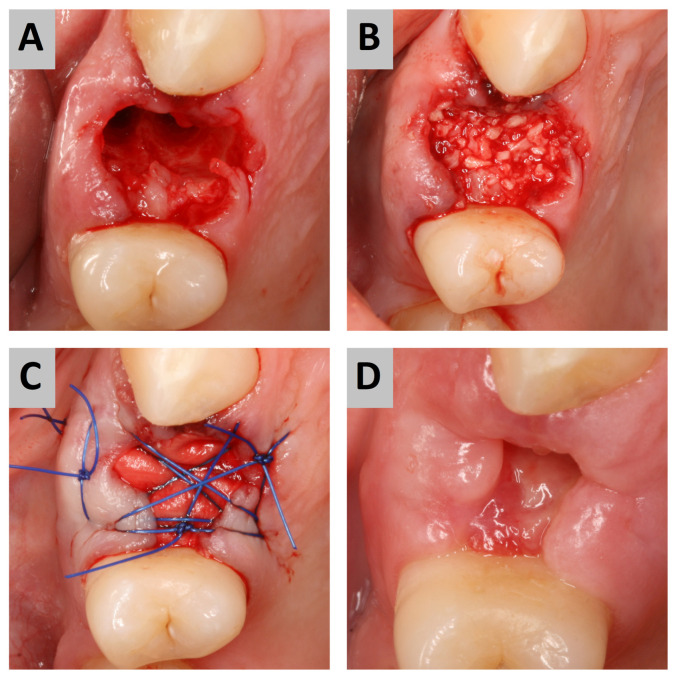
(**A**) Post-extraction socket; (**B**) grafting material inserted into a socket; (**C**) stabilizing a collagen membrane with sutures; (**D**) open healing after 7 days.

Under local anesthesia with 4% articaine with epinephrine (Citocartin 200, Molteni Stomat, Kraków, Poland), an atraumatic intra-alveolar extraction of a first or second maxillary premolar or first or second mandibular molar was performed, taking special care not to damage the buccal bone plate (Figure 3A). The socket was cleared of soft tissue, irrigated with saline, and then filled with an appropriate augmentation biomaterial up to the level of the alveolar crest (Figure 3B). The biomaterial was covered with a collagen membrane (Geistlich Bio-Gide^®^, Geistlich, Wolhusen, Switzerland) and secured with 4-0 nylon sutures using an open healing protocol (Figure 3C). Photographic documentation was taken during the procedure while maintaining the patient’s anonymity.

Postoperative management included the administration of 1 g of amoxicillin or, in cases of allergy, 600 mg of clindamycin every 12 h for 7 days, the application of chlorhexidine gel 3 times daily, and NSAIDs as needed.

A follow-up visit was conducted 7 to 10 days after the procedure. The sutures were removed, photographic documentation was prepared, and a CBCT scan was performed with a field of view (FOV) of 5 cm × 5 cm and a voxel size of 75 µm (Carestream CS 8100 3D) (Figure 3D).

Six months after the procedure, additional CBCT scans with a 5 cm × 5 cm FOV were performed to obtain measurements and plan the implant placement.

### 2.4. Implant Surgery with Trepanobiopsy

**Figure 4:**
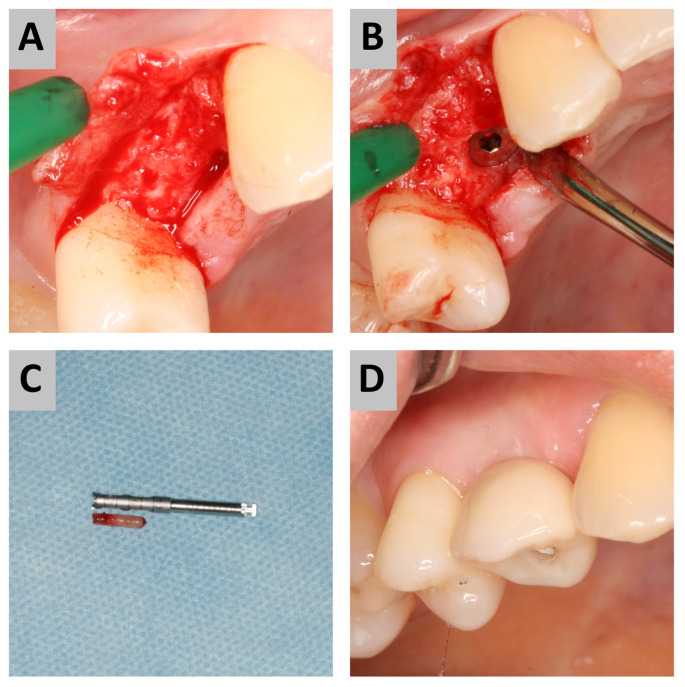
(**A**) Alveolar ridge after 6 months of healing; (**B**) implant secured with a cover screw; (**C**) bone trepanobiopsy; (**D**) screw-retained crown restoration.

Under local anesthesia with 4% articaine with epinephrine (Citocartin 200), a mucoperiosteal flap was elevated, exposing the healed alveolar ridge (Figure 4A). Next, the site was prepared using a sequence of drills, and an implant of the appropriate diameter and length (AB Dental I5, A.B. Dental Devices Ltd, Ashdod, Israel) was placed, achieving primary stability in the range of 20–50 Ncm; the implant slot was then secured with a cover screw using a closed healing protocol (Figure 4B). The use of a trephine with an inner diameter of 2 mm instead of a pilot drill allowed for the collection of a bone biopsy (Figure 4C).

Six months later, the implant was exposed, and a healing screw was placed. After 2–3 weeks of soft tissue healing, an intraoral scan (Dexis IS 3800 W, DEXIS, Quakertown, PA, USA) or a silicone impression on an open tray was taken, based on which a screw-retained crown was fabricated. Following occlusal adjustment, the restoration was delivered to the patient for use (Figure 4D).

### 2.5. Measurement of Alveolar Ridge Dimensions

**Figure 5:**
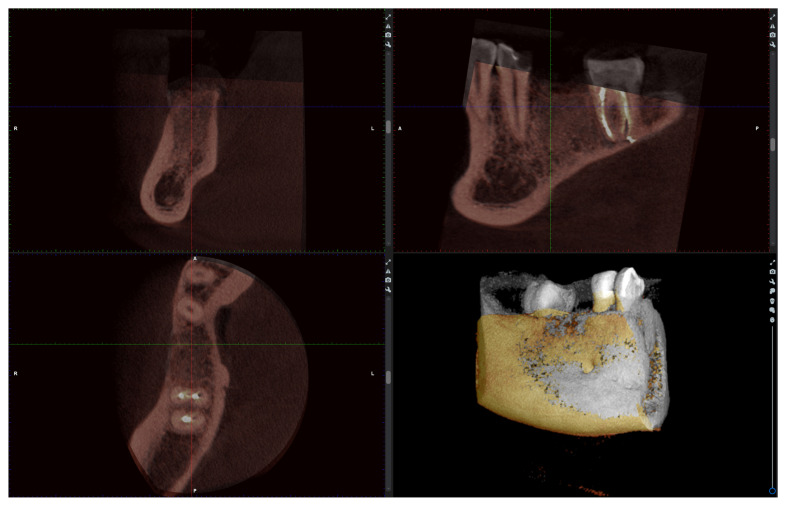
Radiographic superimposition.

**Figure 6:**
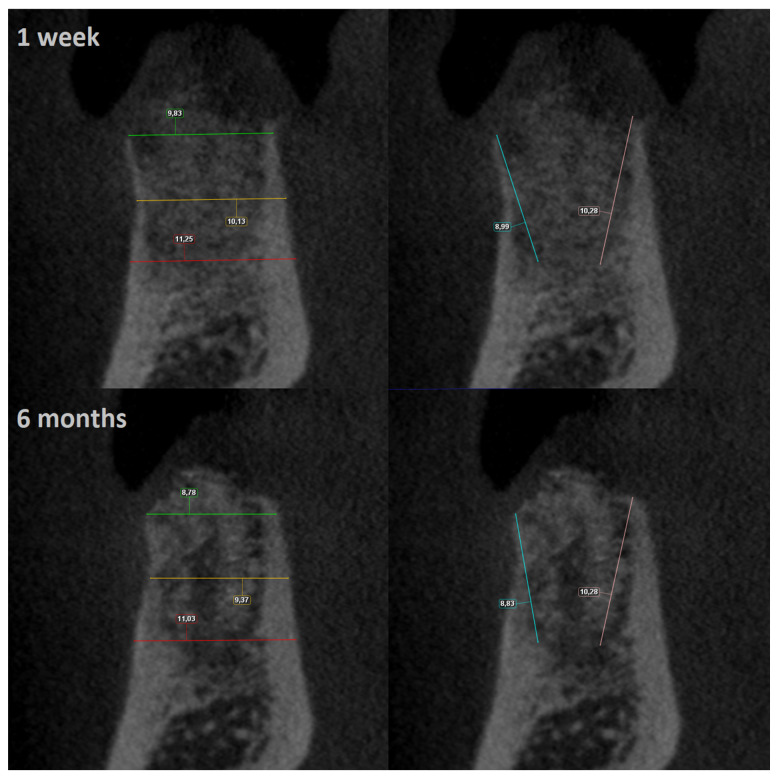
Measurements of alveolar ridge width and height: coronal width (green line), midpoint width (yellow line), apical width (red line), buccal height (blue line), and palatal height (pink line).

CBCT scans performed one week and six months after the procedure were used to conduct radiological measurements. Using Planmeca’s Romexis software (version 6.4.7, Planmeca Oy, Helsinki, Finland), automatic image superimposition was performed after defining three landmarks—most commonly the incisal edges and incisal cusps of adjacent teeth—and the results were then manually corrected (Figure 5). Measurements were taken in the sagittal plane. The sections were oriented so that the buccal bone plate was parallel to the vertical axis. For the premolars group, measurements were taken in the central part of the alveolar socket. For the molars group, measurements were taken in the central part of the alveolar socket of the mesial root. Alveolar socket width measurements were taken at the alveolar crest, at the bottom of the alveolus, and at the midpoint between these points. The height was measured from the bottom of the alveolus to the top of the buccal and palatal or lingual bony plates, respectively (Figure 6).

### 2.6. Radiographic Density Evaluation

ImageJ software (version 1.54p, Wayne Rasband and contributors, National Institutes of Health, Bethesda, MD, USA) was used to determine the radiographic bone density in the augmentation areas.

Density measurements, expressed in Hounsfield units (HU), were performed for each patient on 10 consecutive sagittal cross-sections within representative regions of interest (ROIs).

**Figure 7:**
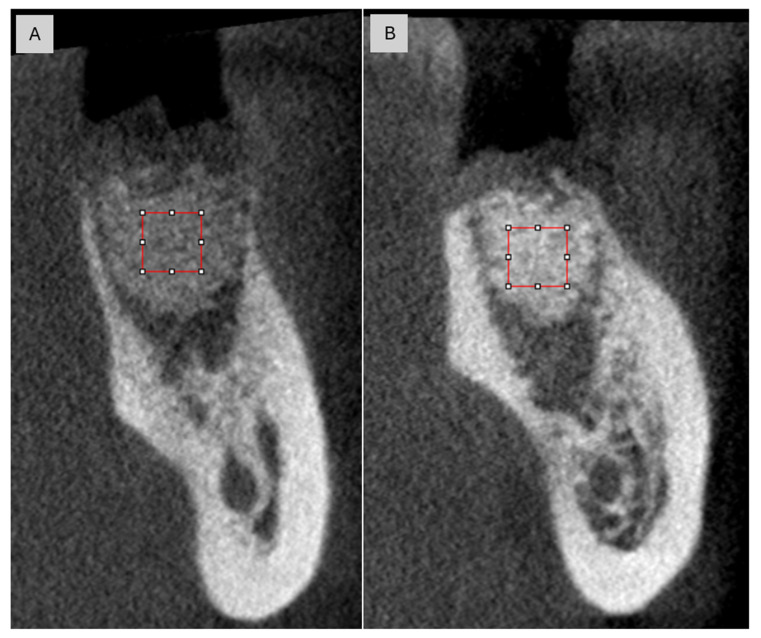
Representative images showing the selection of the region of interest (ROI) for radiographic bone density analysis. (**A**) Sagittal cross-section image obtained 7 days after the procedure with the ROI marked (red square) within the augmented alveolar ridge (area = 3.75 mm^2^). (**B**) Corresponding image obtained 6 months after augmentation, with the ROI placed in the same region and with the same area (3.75 mm^2^) for comparative density analysis.

For each patient, an ROI with a uniform area of 3.75 mm^2^ (a region measuring 50 × 50 pixels) was defined, located in the central part of the alveolar process within the augmentation area. The size of the region of interest was selected to cover the largest possible area at the examined location while maintaining uniform ROI dimensions across all analyzed patients. To improve measurement reproducibility and reduce the influence of local image variability, the final optical density for each patient was calculated as the mean of all 10 consecutive CBCT sections. All measurements were performed by the same investigator according to a standardized protocol. Measurements were performed for each patient at two time points: 7 days and 6 months after the augmentation procedure (Figure 7).

### 2.7. Histological Analysis

Trephine biopsy specimens intended for histochemical analysis were collected during surgical procedures and immediately fixed in a 4% buffered formaldehyde solution. The fixed material was transported to the Department of Histology and Cell Pathology at the Medical University of Silesia in Katowice. Fixation was carried out for 24–48 h. To remove excess fixative, the specimens were rinsed in running tap water for 40 min and subsequently decalcified in a 12% EDTA solution for 7 days.

After decalcification, the samples were dehydrated through a graded series of ethanol solutions, with increasing concentrations from 50% to 99.8%. The specimens were then cleared in intermediate solutions by immersion in a 1:1 mixture of absolute ethanol and xylene for no longer than 15 min, followed by immersion in pure xylene (2 × 10 min) to ensure complete removal of residual ethanol. Subsequently, the tissues were transferred to a 1:1 paraffin–xylene mixture for up to 20 min to partially remove xylene and allow initial paraffin infiltration. In the final step, the specimens were infiltrated with pure paraffin, which completely replaced the remaining xylene, and embedded in paraffin blocks.

All paraffin blocks were sectioned into 5 µm thick slices using a rotary microtome (LEICA RM 2145, Leica Biosystems, Deer Park, IL, USA). The sections were fixed on polysine slides (Menzel-Gläser, Epredia, Dreieich, Germany). Next, the sections were deparaffinized and stained with hematoxylin and eosin (H&E) and Masson’s trichrome using an aniline blue-based commercial kit (BIO 04-010802, Bio Optica, Milano MI, Italy). Masson’s trichrome staining enabled differentiation between non-mineralized collagen (stained blue) and mineralized collagen (stained red), allowing distinction between woven bone and mature lamellar bone.

Histochemical staining analysis was performed using an Eclipse 80i (Nikon, Kyoto, Japan) light microscope.

### 2.8. Statistical Analysis

Performed and obtained measurement results were subjected to statistical analysis with STATISTICA 13.1 software. The dimensional parameters of the alveolar ridge and the radiographic bone density expressed in Hounsfield units (HU) were subjected to statistical analysis. The Shapiro–Wilk test was employed to check the normality of the distribution in the analyzed groups, and Levene’s test was employed to check the equality of variances. When the variables showed a normal distribution and equality of variances, the *t*-test was used to compare the measured parameters at 7 days and 6 months after the socket preservation procedure; otherwise, Wilcoxon’s test was used. Moreover, to compare the studied parameters between the biomaterial groups (BioBank and BioOss), independent *t*-tests and Mann–Whitney U tests were used, respectively.

In the first stage, five dimensional parameters of the alveolar ridge were analyzed: the width at the alveolar crest, at the base of the alveolar socket, and at the midpoint, as well as the height of the buccal and palatal walls. The analysis was conducted separately for two groups of teeth: first and second maxillary premolars and first and second mandibular molars. For each parameter, the change in value after 6 months of healing was determined. Subsequently, the values of these changes were compared between the two augmentation material groups (BioBank and Bio-Oss). All analyzed variables exhibited a normal distribution and equal variances; therefore, Student’s *t*-test for independent samples was used to compare the mean values between the groups.

The analysis of bone radiographic density compared density values at 7 days and 6 months within the same augmentation material, density values between BioBank and BioOss materials at both time points, and changes in density between measurements (ΔHU), calculated as the difference between the density values at 6 months and 7 days. Molars and premolars were analyzed together.

The results are presented as mean values (Mean) along with the standard deviation (SD). A *p*-value of <0.05 was considered statistically significant.

## 3. Results

### 3.1. Alveolar Ridge Radiographic Evaluation

Measurements of width and height taken 7 days and 6 months post-surgery facilitated assessment of changes in these dimensions throughout the healing period. The results are summarized in Table 1 for maxillary first and second premolars and in Table 2 for first and second mandibular molars.

The statistical evaluation revealed no significant differences among the groups for all parameters.

### 3.2. Radiographic Bone Density Measurement

The analysis of bone radiographic density did not account for tooth location (molars and premolars); comparisons were made exclusively between the augmentation biomaterials used.

Bone radiographic density was assessed using values expressed in Hounsfield units (HU) obtained at the augmentation sites 7 days and 6 months after the procedure. In both groups of augmentation biomaterials analyzed, an increase in bone density was observed between the first week and the sixth month post-procedure. This increase was statistically significant in both the BioBank and BioOss groups (Wilcoxon test, *p* = 0.000001).

The mean radiographic density values and standard deviations for both augmentation materials are presented in Table 3.

A comparison of the bone graft materials revealed no significant differences in bone density values between the BioBank and BioOss groups 7 days after the procedure (Mann–Whitney U test, *p* = 0.17).

**Figure 8:**
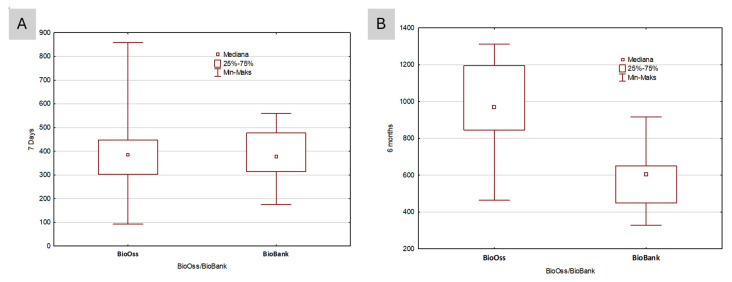
Radiographic bone density (HU) in BioOss and BioBank groups measured: (**A**) 7 days and (**B**) 6 months after augmentation. Box plots represent the median, 25–75% quartiles, and min–max values.

Six months after the procedure, significantly higher bone density values were observed in the BioOss group compared to the BioBank group (Mann–Whitney U test, *p* = 0.000001). The distribution of radiographic density values for both augmentation materials at 7 days and 6 months is shown in Figure 8.

**Figure 9:**
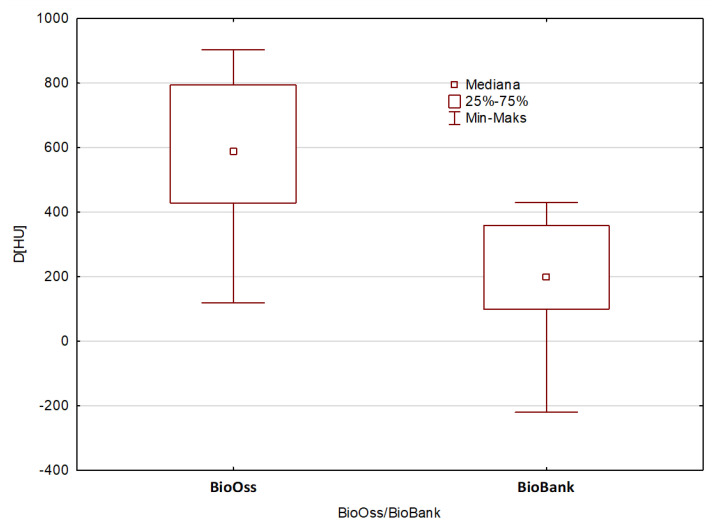
Changes in radiographic bone density (ΔHU) between 7 days and 6 months after augmentation in the BioBank and BioOss groups. Box plots represent median, 25–75% quartiles, and min–max values.

In addition, an analysis of changes in radiographic density between two time points was conducted (ΔHU = HU at 6 months—HU at 7 days). A comparison of ΔHU values between the augmentation materials showed a significantly greater increase in density in the BioOss group than the BioBank group (Mann–Whitney U test, *p* = 0.000001). The distribution of ΔHU values for both biomaterials is presented in Figure 9.

The use of standardized ROI dimensions and averaging measurements across 10 consecutive CBCT sections reduced measurement variability and improved the reproducibility of the optical density analysis.

### 3.3. Histological Evaluation

**Figure 10:**
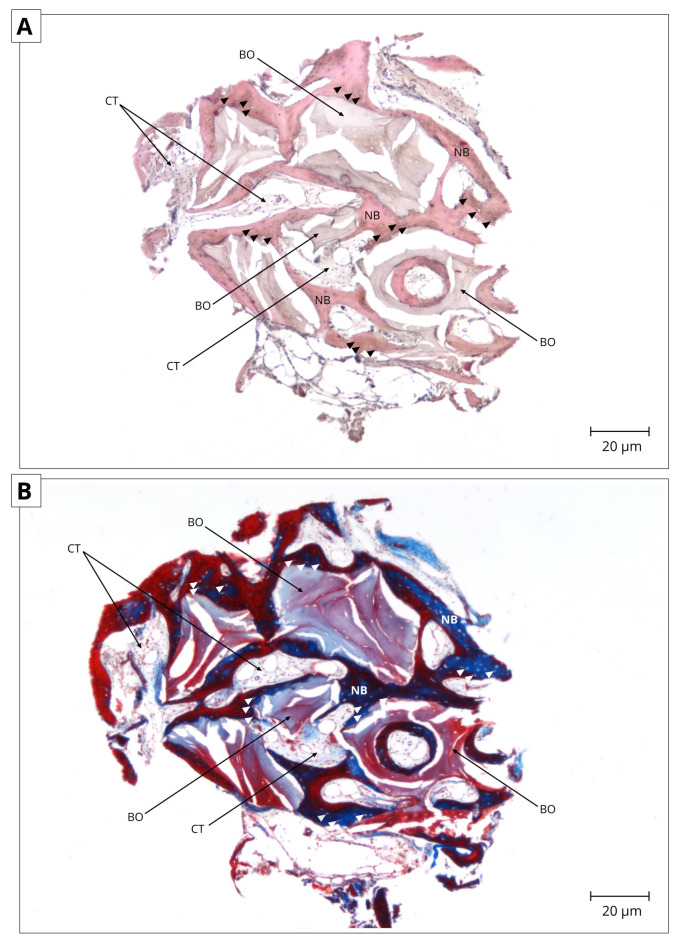
Xenograft group. Two-dimensional (2D) reconstruction of the bone sample at ×40 magnification. (**A**) Hematoxylin and eosin staining; (**B**) Masson’s trichrome staining. Woven bone stained blue and lamellar bone stained red. Residual xenograft particles (BO) surrounded by newly formed bone tissue (NB) with osteocytes (black and white arrows). Connective tissue (CT) consisting of fibroblasts and adipocytes.

**Figure 11:**
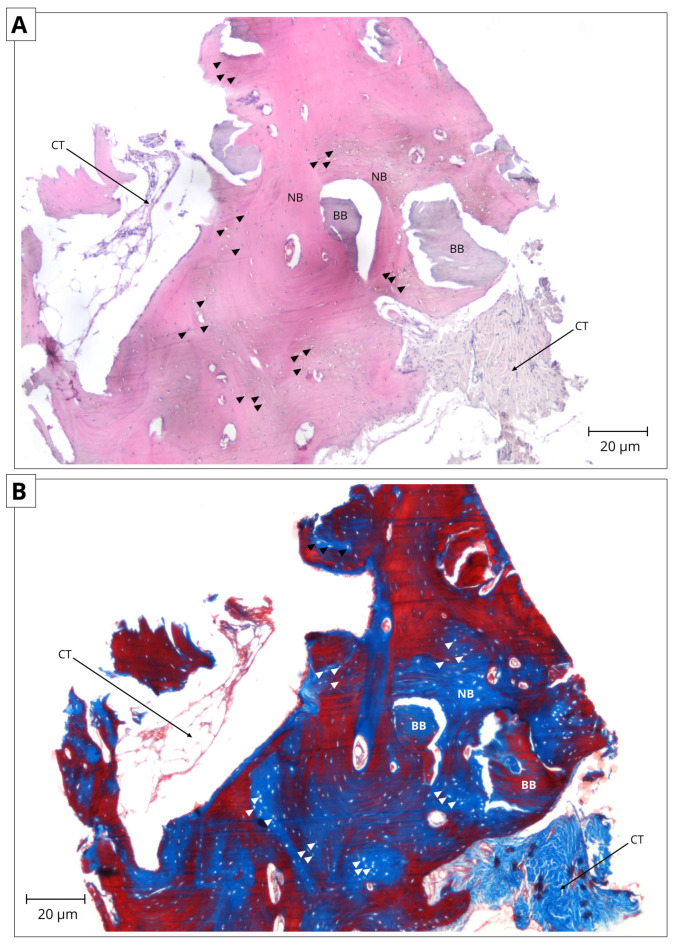
Allograft group. Two-dimensional (2D) reconstruction of the bone sample at ×40 magnification. (**A**) Hematoxylin and eosin staining; (**B**) Masson’s trichrome staining. Woven bone stained blue and lamellar bone stained red. Residual allograft particles (BB) surrounded by newly formed bone tissue (NB) containing osteocytes (black and white arrows). Connective tissue (CT) consisting of fibroblasts and adipocytes.

The subsequent micrographs illustrate specimens stained with hematoxylin and eosin and Masson’s trichrome with aniline blue (Figure 10 and Figure 11). In all 20 trepanobiopsies obtained from patients with xenografts, residual graft material particles were identified on the slides. Conversely, among patients who received an allograft as the augmentation material, residual graft particles were detected in only three of the twenty trepanobiopsies.

In the examination of hematoxylin and eosin staining, new bone tissue in direct contact with the augmentation material was noted in both samples. A structure resembling connective tissue, characterized by a high number of fibroblasts and a lower presence of adipocytes, was also apparent, potentially corresponding to bone marrow. No inflammatory cells were detected. These observations suggest the biocompatibility of both materials.

In Masson-stained sections, samples from both groups exhibited regions of bone characterized by an irregular configuration of collagen fibers, which were stained blue, potentially indicating woven bone. Additionally, areas of bone displayed a regular arrangement of collagen fibers, stained red, likely corresponding to lamellar bone. This finding suggests ongoing remodeling of bone tissue.

## 4. Discussion

The results obtained in the authors’ previous pilot study indicated differences in alveolar socket healing following filling with allogeneic and xenogeneic biomaterials [1]. Alveolar sockets treated with xenograft exhibited greater radiographic bone density after the healing period, a finding also confirmed by the results of this study. However, we did not find evidence to support the superiority of allogeneic material in reducing vertical resorption of the alveolar ridge. This discrepancy may result from the small sample size of the pilot study and from the fact that patients were selected for treatment regardless of buccal bone plate thickness, which is one of the key factors influencing the degree of postoperative bone resorption [50,52,53,55].

Various materials, such as xenografts, allografts, autografts, and alloplasts, can be used in alveolar ridge preservation procedures, and their effectiveness has been confirmed in numerous clinical studies [36,64,65,66,67,68,69]. The effect of the graft type on the volumetric stability of the augmented alveolar ridge has been the subject of numerous research studies.

In their study, Serrano Méndez et al. compared demineralized freeze-dried bone allograft (600–850 μm) (DFDBA) and deproteinized bovine bone mineral xenograft (Bio-Oss Collagen®, 250–1000 μm) (DBBM) in an alveolar ridge preservation procedure [70]. The authors found no statistically significant differences in efficacy between these biomaterials, which is consistent with the results obtained in the present study. Similar results were obtained by Abellán et al., who compared the efficacy of mineralized cortico-cancellous freeze-dried bone allograft (FDBA) (MinerOss®, BioHorizons, Birmingham, AL, USA) and xenogeneic cancellous DBBM materials (Bio-Oss®) in ARP, finding no statistically significant differences in the maintenance of alveolar ridge width and height [71]. Sadeghi et al. also found no statistically significant differences in the reduction in alveolar ridge height and width following alveolar augmentation using DBBM (Bio-Oss®) and cortico-cancellous DFDBA (CenoBone®, Cenobiologics Ltd., Milton Keynes, UK) [72]. These observations are also supported by a meta-analysis conducted by Natto et al., which found no statistically significant difference in the efficacy of xenogeneic and allogeneic materials in ARP [73].

Another factor that may influence the effectiveness of the material used for socket preservation is its degree of mineralization. In this study, a partially demineralized allogeneic cortico-cancellous material was used to ensure volumetric stability of the graft while enabling proper remodeling into bone tissue. In a study by Demetter et al. comparing the efficacy of FDBA derived from cancellous bone, cortical bone, and a 1:1 combination, the authors found no statistically significant differences in the dimensional stability of the augmented alveolar ridge [74]. Wood and Mealey compared the effectiveness of DFDBA and FDBA in ARP procedures and found no statistically significant differences in the preservation of alveolar ridge dimensions [75].

A characteristic of xenogeneic material is its slow resorption, which may result in a higher proportion of residual graft in the newly formed bone tissue [76,77]. The results regarding this issue are inconclusive. Sadeghi et al. demonstrated a higher residual graft content in alveolar sockets filled with DBMM than in those filled with DFDBA [72]. Molly et al. demonstrated a statistically significantly higher residual graft content following ARP in the bovine xenograft group than in the other materials tested [78]. Zampara et al. compared xenogeneic, allogeneic, and alloplastic materials in terms of residual graft content following ARP procedures, finding a statistically higher proportion of biomaterial particles in biopsy samples for the xenogenic material [79]. However, some researchers found no difference in residual graft content among different biomaterials [70,71]. In this study, we did not determine the percentage of residual graft in the biopsy specimens; however, the fact that xenograft particles were detected in all collected samples, whereas allograft particles were found in only three of the twenty samples, may indicate faster remodeling of the allogeneic material. These observations are supported by a meta-analyses conducted by Stumbras et al. and Chan et al. on grafts used in ARP, in which DFDBA showed the lower amount of residual graft particles after socket preservation then xenogeneic materials [13,48].

In their systematic review of the use of xenogeneic materials in ARP, Khan et al. demonstrated that the residual graft content may also depend on the xenograft’s species of origin [80]. Madi et al. compared two xenogeneic bovine materials and found a statistically significant difference in residual graft content [81]. Guarnieri et al. compared bovine and porcine xenografts and found no difference in residual graft content [82].

The biomaterial may consist of cortical bone, cancellous bone, or a mixture of cortical and cancellous bone. Demetter et al. demonstrated a statistically significantly higher proportion of residual allograft of cortical origin compared to cancellous bone and a 1:1 mixture of cortical and cancellous bone [74]. Eskow and Mealey also demonstrated a statistically higher residual graft content in cortical FDBA compared to cancellous FDBA [83]. Soardi et al. obtained contrary results, finding no statistically significant difference in residual graft content between cortical and cancellous allografts [84].

One way to reduce the residual xenograft content in the tissue may be to combine it with an allograft during the procedure. Shaikh et al. and Serrano et al. demonstrated that ARP using a 1:1 mixture of xenograft and allograft is adequate for proper implanto-prosthetic treatment [85,86].

The studies cited above indicate that the issue of residual graft and its impact on the success of implant treatment warrants further investigation. Many authors have demonstrated the success of implant treatment performed in augmented bone [42,87,88,89,90,91,92,93,94].

As a result of the ARP procedure, the alveolar socket is filled with a mixture of graft material and blood clot. If primary closure is not achieved, this mixture is exposed to the oral environment. However, recent studies indicate that primary wound closure does not improve the outcomes of socket preservation procedure and may, at the same time, lead to soft tissue complications.

Seo et al. found no statistically significant differences between primary and secondary healing in ARP procedures in terms of the effectiveness of preserving the dimensions of the alveolar ridge and the histological composition of the bone tissue at the augmentation site [95]. Similarly, Kim et al. reported a similar percentage of vital bone and residual graft in cases of primary and secondary healing [96]. Aladmawy et al. found no differences in the preservation of alveolar ridge dimensions between the primary and secondary healing groups; however, in the primary healing group, there was a statistically significant reduction in the width of the keratinized gingiva at the surgical site [97]. In their studies, Wei et al. compared socket preservation procedures with primary and secondary healing, where open healing had a more favorable effect on the preservation of the keratinized tissue width, while the efficacy in terms of alveolar ridge dimensions preservation, bone histological composition, and one-year implant treatment outcomes was similar [98,99]. These observations are also supported by a study conducted on dogs by Choi et al., who demonstrated comparable efficacy of ARP procedures using open and closed healing, with a lesser reduction in the width of the keratinized gingiva in the primary healing group [100].

Although socket preservation procedures using only augmentation material can provide an adequate therapeutic effect, the use of barrier membranes offers many benefits.

In their study, Perelman-Karmon et al. demonstrated a higher percentage of vital bone in bone biopsies taken from alveolar sockets augmented with DBBM and covered with a resorbable membrane than in alveolar sockets filled with DBBM and secured only with sutures [101]. Lim et al. evaluated the efficacy of ARP procedures using DBBM alone and DBBM combined with a collagen membrane, compared with natural healing of the alveolar socket [102]. The authors demonstrated that the use of a xenograft combined with a membrane yielded the best results in preserving the dimensions of the alveolar ridge. Meta-analyses and systematic reviews also indicate the beneficial effects of using barrier membranes in alveolar ridge preservation procedures [16,103,104].

Collagen membranes are characterized by their ability to resorb over a period of 1 to 38 weeks, depending on the manufacturing process and materials used [105]. As a result of secondary healing, the membrane is exposed to the oral environment, which leads to its faster degradation [106]. These observations have led to attempts to use new types of membranes, namely cross-linked membranes with a longer resorption time, as well as new surgical techniques, such as the double-layer membrane technique [107,108,109,110,111]. Pilot studies conducted by Friedmann et al. indicate the effectiveness of cross-linked membranes in open healing during socket preservation [109]. Animal studies conducted by Tal et al. and Jin et al. indicate that cross-linked and non-cross-linked membranes are similarly effective, including in the case of secondary healing [110,111]. The study by Choi et al. did not demonstrate statistically significant differences in the efficacy of ARP with secondary healing using a double-layer membrane technique compared to a single-layer membrane [108].

Evidence from guided bone regeneration and ARP shows similar clinical outcomes when resorbable (collagen) and non-resorbable (dPTFE/e-PTFE) membranes are compared. A systematic review conducted by Patil et al. indicates that resorbable and non-resorbable membranes are equally effective in guided bone regeneration procedures [112]. Arbar et al. compared the use of collagen and PTFE membranes in ARP procedures with open healing, finding no significant differences in the effectiveness in maintaining the dimensions of the alveolar ridge or in the quality of bone tissue in the augmented area [113]. The efficacy of socket preservation using collagen and dPTFE membranes was also assessed by Lin et al., who found no significant differences in terms of keratinised tissue width, bone density, insertion torque, and the effectiveness of preserving alveolar ridge dimensions [114]. Nevertheless, employing a dPTFE membrane resulted in a significantly higher vertical mucosal thickness.

In this study, we observed no complications in the healing of alveolar sockets following an open-healing protocol using a single-layer, non-cross-linked collagen membrane. These findings are supported by a meta-analysis conducted by López-Valverde et al., which demonstrates the efficacy of ARP procedures using a collagen membrane [115]. It should be noted, however, that suboptimal oral hygiene may increase the risk of infection in the augmented area. For this reason, the use of chlorhexidine- or propolis-based products is recommended during the postoperative period [116,117].

Socket preservation procedures may affect the density of the new bone tissue forming at the augmented site. Khan et al. found a statistically significant increase in radiographic bone density following ARP procedures using DFDBA and PRF compared with natural healing [34]. In their research, López Sacristán observed that autologous tooth-derived grafts produced higher CBCT Hounsfield units than ungrafted sockets [118]. Ma et al. demonstrated that autologous concentrated growth factors alone in extraction sockets led to higher bone mineral density [119]. Kloss et al. demonstrated that the addition of hyaluronic acid to an allograft in socket preservation procedures can lead to an increase in radiographic bone density [120].

Bone mineral density may influence many factors relevant to implant treatment. Ivanova et al. in their studies assessed the effect of bone density following ARP procedures using various augmentation biomaterials on primary and secondary implant stability, as well as the percentage of viable bone in tissue biopsies taken during implantation. The authors demonstrated a statistically significant positive correlation between radiographic density and the parameters described [121,122]. Farré-Pagés et al., Khan et al., Haghanifar et al., Isoda et al., Padmaja and Rajasekar, Merheb et al., Mikic et al., Rostamzadeh et al. also showed a positive correlation between radiographic bone density and primary implant stability [123,124,125,126,127,128,129,130]. In their meta-analysis, Putura et al. highlight a significant correlation between bone density and primary implant stability [29].

In this study, the xenogeneic material demonstrated a statistically significantly higher bone mineral density after the healing period compared to the allograft, although this may be due to a higher residual graft content. This characteristic may be important when selecting a biomaterial for ARP procedures in cases of planned implant placement.

Masson’s trichrome staining method allows for the differentiation of less mineralized, younger woven bone from mature, more mineralized lamellar bone [131,132,133,134]. Flifl et al. conducted a study on rabbits in which the use of Masson’s trichrome staining at 2 and 6 weeks after augmentation of controlled bone defects filled with bone substitute biomaterials enabled assessment of the ossification process in the augmented areas [135]. In the previous pilot study, the allograft group exhibited areas of both woven and lamellar bone, whereas the xenograft group exhibited mainly areas of woven bone, indicating faster bone maturation following allograft application [1]. Subsequent studies did not confirm these observations, as biopsies from the allograft and xenograft groups showed varying amounts of areas with lower and higher mineralization. These differences may be explained by a larger study group, greater experience of the histologist conducting the study, or a multitude of factors that could influence bone tissue mineralization. These observations are purely qualitative, and no quantitative measurements were performed.

Histological analysis of the specimens did not reveal inflammatory cells in the tissues surrounding the augmented area. This is consistent with the results obtained by other researchers [26,78,134]. Nevertheless, some studies indicate the presence of small numbers of inflammatory cells in the vicinity of the graft [81,136,137].

## 5. Limitations of the Study

Despite the prospective and randomized design, this study has several limitations. First, the sample size, although adequate for statistical significance in primary outcomes, remains relatively small, which may limit the detection of subtle differences between the biomaterials. Second, the study focused on specific tooth types (maxillary premolars and mandibular molars); therefore, the results may not be directly generalizable to other anatomical regions with different bone densities or ridge morphologies. Third, a significant technical limitation involves the assessment of bone density using CBCT. Unlike multislice computed tomography, CBCT does not provide standardized Hounsfield Units (HU), as the gray values are influenced by factors such as field of view, scatter radiation, and the positioning of the object within the scan area [138,139]. This lack of absolute calibration can lead to variability in density measurements across different devices and protocols, making direct comparisons between studies challenging [140,141,142]. Fourth, despite randomization, inherent patient-related variability may have influenced the outcomes. Factors including age, sex, systemic health status, smoking habits, oral hygiene compliance, buccal bone plate thickness at the time of extraction, and individual wound healing capacity can all affect bone regeneration and graft incorporation. While inclusion and exclusion criteria were applied to minimize confounding, the relatively small sample size may not have been sufficient to fully balance all potential confounders across groups. Fifth, the histological analysis in this study was limited to qualitative evaluation using hematoxylin and eosin (H&E) and Masson’s trichrome staining, without quantitative histomorphometric measurements. This approach does not provide objective data on the percentage of newly formed bone, residual graft material, or connective tissue within the biopsy specimens. Since no residual graft was detected in most of the samples from the allograft group, we decided not to perform histomorphometric analyses. Finally, the 6-month healing period provides insights into early remodeling, but long-term data on the stability of the augmented ridge following implant loading are still required.

## 6. Conclusions

Within the limitations of this prospective randomized clinical study, the two materials demonstrated comparable effectiveness in preserving alveolar ridge height and width. Throughout the 6-month healing period, both materials exhibited a statistically significant increase in radiographic density. The xenograft group displayed a significantly greater increase than the allograft group. Furthermore, our histological analysis indicated a more rapid remodeling of the allograft into native bone. In summary, both biomaterials proved appropriate for alveolar ridge preservation.

## Figures and Tables

**Table 1:** A comparison of alveolar ridge width and height alterations, in millimeters, after 6 months of healing in the maxillary first and second premolars group.

Clinical Parameter	Allograft		Xenograft		
	Mean (Sd.)	Median (IQR)	Mean (Sd.)	Median (IQR)	*p*-Value
Horizontal width alteration coronally	1.18 (0.66)	0.97 (1.12)	1.06 (0.69)	0.90 (0.68)	0.56
Horizontal width alteration midpoint	0.70 (0.40)	0.97 (0.45)	0.57 (0.53)	0.60 (0.90)	0.83
Horizontal width alteration apically	0.27 (0.81)	0.38 (0.54)	0.41 (0.37)	0.45 (0.60)	0.92
Vertical height alteration buccally	0.76 (0.36)	0.62 (0.24)	1.26 (0.56)	1.41 (0.45)	0.49
Vertical height alteration palatally	0.55 (0.62)	0.46 (0.61)	0.67 (0.39)	0.72 (0.23)	0.27

*p*-value—independent *t*-test.

**Table 2:** A comparison of alveolar ridge width and height alterations, in millimeters, after 6 months of healing in the mandibular first and second molars group.

Clinical Parameter	Allograft		Xenograft		
	Mean (Sd.)	Median (IQR)	Mean (Sd.)	Median (IQR)	*p*-Value
Horizontal width alteration coronally	1.59 (0.21)	1.51 (0.37)	2.09 (0.66)	2.32 (1.05)	0.65
Horizontal width alteration midpoint	0.39 (0.27)	0.38 (0.15)	0.48 (0.08)	0.45 (0.08)	0.79
Horizontal width alteration apically	0.16 (0.33)	0.00 (0.00)	0.09 (0.12)	0.00 (0.15)	0.48
Vertical height alteration buccally	1.01 (0.94)	0.67 (0.97)	0.50 (0.40)	0.45 (0.22)	0.55
Vertical height alteration lingually	1.31 (0.63)	1.65 (0.37)	0.77 (0.57)	0.63 (0.67)	0.48

*p*-value—independent *t*-test.

**Table 3:** The mean radiographic bone density levels in Hounsfield units.

Material	1 Week		6 Months		
	Mean	Sd.	Mean	Sd.	*p*-Value
Allograft (BioBank)	390.40	104.95	582.84	156.69	0.000001
Xenograft (BioOss)	389.58	183.88	979.85	231.37	0.000001

*p*-value—Wilcoxon test.

## Data Availability

The original contributions presented in the study are included in the article; further inquiries can be directed to the corresponding author.
